# Nitrous Oxide sedation for intra-articular injection in juvenile idiopathic arthritis

**DOI:** 10.1186/1546-0096-6-1

**Published:** 2008-01-15

**Authors:** Yosef Uziel, Gil Chapnick, Michal Rothschild, Tsivia Tauber, Joseph Press, Liora Harel, Philip J Hashkes

**Affiliations:** 1 Divisions of Rheumatology, Department of Pediatrics, Meir Medical Center, Kfar Saba, Tel Aviv University, Israel; 2 Divisions of Rheumatology, Department of Pediatrics, Asaf-Harofe Medical Center, Tsrifin; Tel Aviv University, Israel; 3 Divisions of Rheumatology, Department of Pediatrics, Soroka University Medical Center, Beer-Sheva; Israel; 4 Divisions of Rheumatology, Department of Pediatrics, Schneider Medical Center, Petah- Tikva, Tel Aviv University, Israel; 5 Section of Pediatric Rheumatology, Department of Rheumatic Diseases, Cleveland Clinic, USA

## Abstract

**Background:**

Intra-articular corticosteroid injection in juvenile idiopathic arthritis (JIA) is often associated with anxiety and pain. Recent reports advocate the use of nitrous oxide (NO), a volatile gas with analgesic, anxiolytic and sedative properties.

**Objective:**

To prospectively evaluate the effectiveness and safety of NO analgesia for intra-articular corticosteroid injection in JIA, and to assess patients and staff satisfaction with the treatment.

**Methods:**

NO was administered to JIA patients scheduled for joint injection. The patient, parent, physician and nurse completed visual-analog scores (VAS) (0–10) for pain, and a 5-point satisfaction scale. Change in heart rate (HR) during the procedure was recorded in order to examine physiologic response to pain and stress. Patient's behavior and adverse reactions were recorded.

**Results:**

54 procedures (72 joints) were performed, 41 females, 13 males; 39 Jewish, 13 Arab; mean age was 12.2 ± 4.7 year. The median VAS pain score for patients, parents, physicians and nurses was 3. The HR increased ≥ 15% in 10 patients. They had higher VAS scores as evaluated by the staff. The median satisfaction level of the parents and staff was 3.0 and 5.0 respectively. Adverse reactions were mild.

**Conclusion:**

NO provides effective and safe sedation for JIA children undergoing intra-articular injections.

## Introduction

Intra-articular corticosteroid injection is one of the cornerstones of treatment for children with juvenile idiopathic arthritis (JIA), particularly oligoarthritis [1]. Major side effects are few except for the pain and the anxiety that accompanies the procedure.

Several techniques are used in an effort to reduce pain including conscious sedation using intravenous benzodiazepine or general anesthesia [2]. Recently the use of nitrous oxide (NO) as a safe and effective sedation method has been reported [3-6]. NO is a volatile gas with analgesic, anxiolytic and sedative properties, mixed together with oxygen. NO has been used for more than 200 years [7].

Few studies have reported on the safety and efficacy of NO in JIA. Cleary et al, showed that a fixed mixture of oxygen and NO, given using a self-delivery device was safe and effective for intra-articular injection in JIA [8].

Using NO by continuous flow method facilitates its' use in younger patients, and can also enable the administration of variable NO concentrations up to 70% [3].

Our study aim was to evaluate the effectiveness and safety of NO analgesia for intra-articular corticosteroid injections in JIA, and to assess patients and staff satisfaction with the treatment.

## Patients and methods

NO was administered to healthy JIA patients older than 3 years of age who were scheduled for joint injection, and whose parents consented to participate in the study. Since this procedure requires cooperation patients younger than 3 years were not included in this study. They were injected under general anesthesia. The institutional ethics committee approved the study.

There was no comparison group. The apparatus used to administer NO was the MDM- Matrx N_2_0 mixer^® ^that allows various concentration of NO to be used with a minimum O_2 _level of 30%, and also has a non-rebreathing circuit. NO was administered in the pediatric day unit by a specially assigned pediatrician who underwent formal training for sedation and a PALS course. Oxygen saturation, heart rate (HR), and respiratory rate were all constantly monitored and recorded by a team member not participating in the injection itself. NO concentration was gradually increased up to a level of 30–50% that was then maintained during the entire procedure (no age related differences in NO concentration). EMLA (AstraZeneca, Wilmington, DE) was placed in all patients at least one hour before the procedure. Patients were not restrained, and when possible the mask was self held. At the end of the procedure 100% oxygen was administered for 3–5 minutes.

Assessment of the procedural pain was made by the patient, parent(s), participating nurse and physician using an age specific visual analog score (VAS) with a scale of 0–10 [9]. The satisfaction of the parents and staff was also assessed by a Likert scale, with a score of 0–5 (5 is best).

All adverse effects were recorded and scored, as was patient behavior during the procedure.

### Statistics

Data analysis was performed using standard software (Sigmastat). One-way analysis of variance on ranks was used to compare between VAS scores of the patients, parents and staff. Rank sum test was used to compare the patients with and without a significant increase in HR during the procedure.

## Results

54 procedures (72 joints) were performed in 43 patients, 31 females, 12 males; 32 Jewish, 11 Arab; mean age was 12.5 ± 4.8 years (range 3.7–18). Twenty of the patients were under 10 years of age and twelve were under age 7 years. Six patients had 2 separate procedures, one had 3, and another had 4 procedures. The majority were injections of large lower extremity joints, only 3 were of small joints of the hands.

**Figure 1:**
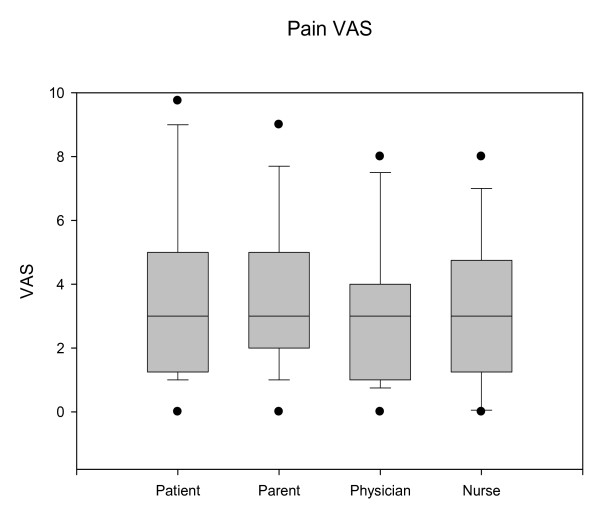
**Pain VAS**. Median pain VAS for the patients, parents, physicians and nurses. VAS- Visual analog scale.

All parents, 93% of physicians and 89% of nurses reported that NO eased the child's pain, and made the procedure easier to perform. The median pain VAS for the patients, parents, physicians and nurses was 3 (Figure 1). There were no significant differences between the different reporter groups. There was no difference in the VAS score between older and younger children.

The HR increased ≥ 15% in 10 patients. These patients had significantly higher VAS as assessed by the physicians (p = 0.006) and nurses (p = 0.010) (Table 1). Nurses and physicians were satisfied with the treatment (Table 2, median level = 5.0). Parents were slightly less satisfied with a median satisfaction level of 3.0 (Table 2). When we compared the VAS between the first and the following injections for those patients who had several injections, no significant differences were found. Recovery time from the procedure was immediate. Adverse reactions were mild, vomiting in 2 patients and shivering in one patient that resulted in discontinuation of NO. No patient had a decrease in oxygen saturation levels. No other severe adverse events were observed.

**Table 1:** Pain VAS in the low vs. high HR change groups.

	**Patient VAS**	**Parent VAS**	Physician VAS*	Nurse VAS*
**Low ΔHR**	2.75	3.00	2.25	3.00
**High ΔHR**	5.50	5.00	4.50	5.00

VAS: Visual analog scale; HR: heart rate

*P for physicians- (p = 0.006) and nurses (p = 0.010) between the low and high ΔHR groups

**Table 2:** Procedure satisfaction

	**Parent**	**Physician**	**Nurse**
Median Likert Score	3	5	5
Mean Likert Score ± SD	3.0 ± 2.4	4.1 ± 1.3	4.3 ± 1.2

Likert score of 0–5 (5 is best)

SD: Standard deviation

## Discussion

Our study reemphasizes the efficacy of NO as an optional sedation for JIA intra-articular injections with low VAS scales assigned by patients, parents and the medical staff. We found a good correlation between the VAS recorded by the medical staff and objective physiological parameters such as the increase in the HR. This finding can be used for better pain monitoring during the procedure.

Children with JIA suffer from pain from procedures including joint injections, and pain related to their arthritis. We offer this procedure for all children, even adolescents, as NO is easy to use in our ambulatory service. Effectively reducing procedural pain can reduce the anxiety with each procedure which itself can exacerbate arthritis pain [10]. Pain reduction is increased if undertaken from the first painful procedure. Among children with newly diagnosed cancer, those who had inadequate analgesia during the first bone marrow aspiration or lumbar puncture had significantly increased distress during subsequent procedures when compared to children who received analgesia during the first procedure [11].

Relaxation before and a comfortable atmosphere during sedation, make the procedure easier to perform. It is possible that better relaxation techniques used by Cleary et al. explain their lower VAS scores (median VAS of 1, mean of 2.1) than in our study [2]. Cleary et al frequently used a play specialist prior to the procedure (personal knowledge). While not examined in this study it is also possible that their lower scores are partially explained by temperament differences between their British subjects and our subjects. In our study there was no significant difference between Arab and Jewish patients.

In the group of children who underwent more than one procedure (N = 8) we did not demonstrate less pain or stress in subsequent injections despite our assumptions from the childhood cancer data [11]. The small sample size of this subgroup was not of sufficient power for this analysis.

As in other studies [5,11,12], NO was safe with no major side effects. When comparing NO to other sedation options such as IV midazolam or general anesthesia, NO stands as a safer option. Moreover, midazolam has no analgesic property so for painful procedures it is commonly administered together with an opoid, a practice that may decrease midazolams' safety profile [13]. It is also easier to schedule a patient for NO sedation as compared to the use of general anesthesia.

## Conclusion

NO provides an effective and safe sedation for children with JIA undergoing intra-articular injections.

## Competing interests

The author(s) declare that they have no competing interests.

## Authors' contributions

YU – senior author – participated in the study design, coordination of the study, and writing the manuscript.

GC – participated in the design of the study and performed the statistical analysis, and helped to draft the manuscript.

MR – participated in the design of the study, coordination data collection, and helped to draft the manuscript.

TT – participated in the design of the study, data collection, and helped to draft the manuscript.

JP – data collection, and helped to draft the manuscript.

LH – data collection, and helped to draft the manuscript.

PJH – participated in the design of the study, and writing the manuscript.

All authors participated in writing and reading the manuscript and approved the final version of the manuscript.
